# Development of a 12-Valent HPV L1 Virus-like Particle Vaccine Using an Enhanced Baculovirus Expression System

**DOI:** 10.3390/vaccines14050398

**Published:** 2026-04-29

**Authors:** Jae-Deog Kim, Eun-Ha Kim, Ji-Hoon Lee, Seong-Yeong Kim, Jong-Min Oh, Yerae Cho, Hyunil Kim, WonSeok Gwak, Soo-Dong Woo, Beom-Ku Han, Jae-Bang Choi

**Affiliations:** 1 Optipharm Inc., Cheongju 28158, Republic of Korea; kimjd@optipharm.co.kr (J.-D.K.); keunha@optipharm.co.kr (E.-H.K.); leejih@optipharm.co.kr (J.-H.L.); ksyoung@optipharm.co.kr (S.-Y.K.); ojm@optipharm.co.kr (J.-M.O.); choyr@optipharm.co.kr (Y.C.); hikim@optipharm.co.kr (H.K.); 2 Division of Clinical Vaccine Research, Center for Vaccine Research National Institute of Infectious Diseases, National Institute of Health, Korea Disease Control and Prevention Agency (KDCA), Cheongju 28160, Republic of Korea; wsgwak@korea.kr; 3 Department of Agricultural Biology, College of Agriculture, Life & Environment Science, Chungbuk National University, Cheongju 28644, Republic of Korea; sdwoo@cbnu.ac.kr

**Keywords:** HPV, vaccine, VLP, enhanced baculovirus expression vector system

## Abstract

**Background/Objectives**: Cervical cancer, predominantly driven by persistent infection with high-risk human papillomaviruses (HPVs), is one of the most common malignancies and an important cause of cancer-related mortality among women worldwide. Although existing licensed prophylactic HPV vaccines confer excellent protection, their global use remains suboptimal due to concentrated manufacturing capacity and high production costs. This study aimed to establish a cost-effective multivalent HPV virus-like particle (VLP) vaccine platform. Specifically, we used an enhanced baculovirus expression vector system to produce a 12-valent HPV VLP vaccine to improve antigen yield, thereby reducing manufacturing costs and ultimately improving affordability and availability in low- and middle-income countries. **Methods**: Optimized expression cassettes and an insect cell culture process were designed to enhance productivity across 12 HPV L1 genotypes. A scalable purification scheme integrating ion-exchange and adsorption chromatography was developed to produce high-purity VLPs with consistent structural integrity. Immunogenicity was assessed in a murine model. Elicited HPV type-specific IgG antibody responses were compared with those induced by the licensed 9-valent HPV vaccine. **Results**: The assembled 12-valent VLPs were comprehensively characterized using biophysical and immunochemical analyses, confirming structural stability and correct antigenicity. In vivo immunogenicity studies in mice showed strong and serotype-specific IgG responses, comparable or superior to those induced by the licensed 9-valent commercial vaccine. **Conclusions**: The enhanced baculovirus expression vector system is a versatile and economically sustainable platform for next-generation HPV vaccine production. This technology offers a promising approach to lowering vaccine manufacturing costs and improving global access, particularly in low- and middle-income regions heavily burdened by HPV-associated diseases.

## 1. Introduction

Cervical cancer, a malignant tumor, arises primarily from the epithelial and mucosal tissues of the cervix and is among the most common cancers in women worldwide [1,2]. Nearly all cases of cervical cancer harbor human papillomavirus (HPV) DNA, and persistent HPV infection is a major etiological factor underlying its development [3]. To prevent HPV-associated cervical cancer, several prophylactic HPV vaccines have been licensed, including Cervarix^®^, GARDASIL^®^, GARDASIL^®^9, and Cecolin^®^ [4]. Cervarix^®^ and Cecolin^®^ are bivalent vaccines targeting HPV types 16 and 18, whereas GARDASIL^®^ is a quadrivalent vaccine targeting types 6, 11, 16, and 18. GARDASIL^®^9 is a nonavalent vaccine containing the same four HPV types as GARDASIL^®^ plus five additional high-risk types (31, 33, 45, 52, and 58), thereby broadening protection against cervical cancer [5,6].

The International Agency for Research on Cancer (IARC) classifies HPV types 16, 18, 31, 33, 35, 39, 45, 51, 52, 56, 58, and 59 as carcinogenic to humans (Group 1 high-risk types) in 2009 [7,8]. Currently, the nonavalent vaccine GARDASIL^®^9 is the multivalent HPV vaccine covering the largest number of types, but it does not include HPV types 35, 39, 51, or 59 [6]. Recently, an investigational 14-valent HPV vaccine that includes two low-risk types (6 and 11) in addition to these high-risk types has been evaluated for immunogenicity; however, this candidate has not yet been licensed for commercial use [9].

HPV L1 capsid proteins self-assemble into VLPs in various recombinant expression systems [10]. Owing to their highly ordered, repetitive surface geometry, VLP-based vaccines can efficiently elicit robust T-cell responses and, by displaying multiple copies of conformational epitopes, induce strong B-cell activation and antibody production [11,12]. All currently licensed prophylactic HPV vaccines are based on L1 VLPs, and their safety and high preventive efficacy have been demonstrated in clinical trials and post-licensure studies [13,14].

More than 80% of the global burden of HPV-related cervical cancer occurs in low- and middle-income countries (LMICs) [15,16]. Despite this disproportionate burden, currently licensed HPV vaccines have been introduced into the national immunization programs (NIPs) of only around 60% of WHO Member States, and an estimated 60% of all HPV vaccine doses supplied by 2025 are projected to be procured through the public sector [17,18]. In Gavi-eligible countries, these public-sector vaccines are typically supplied at a subsidized, tiered price of approximately USD 4.5 per dose via Gavi and UNICEF procurement mechanisms [1,8,17]. However, several low-income countries have reported that even this public-sector price is difficult to sustain, and in some Gavi-ineligible LMICs, HPV vaccine introduction has been delayed or deemed infeasible primarily because of cost constraints [16]. Consequently, cost-effectiveness and long-term financial sustainability are critical considerations for public-sector HPV vaccine adoption and scale-up [18].

Multivalent HPV vaccines designed for public-sector use inevitably raise concerns about increased manufacturing costs associated with broader type coverage. Nevertheless, recent economic evaluations suggest that gender-neutral vaccination with the 9-valent HPV vaccine can reduce the long-term public health burden of HPV-related diseases and is cost-effective compared with girls-only vaccination using a quadrivalent vaccine [1]. In this context, low-cost HPV vaccines produced using platforms such as enhanced baculovirus expression vector systems align well with the goals of WHO, Gavi, and developing-country vaccine manufacturers (DCVMs), ensuring equitable and sustainable HPV vaccine supply.

Baculovirus expression vector systems (BEVSs) are widely used to produce diverse recombinant antigens [19,20]. Owing to their high productivity and ability to perform many eukaryotic post-translational modifications, BEVSs are particularly well suited for the manufacturing of structurally complex proteins such as VLPs [21]. Insect cells and larvae are the principal hosts for BEVS-based production, and the excellent safety profile of baculoviruses in humans has supported the licensure of several BEVS-derived therapeutics and vaccines [20]. Previously, we developed a high-expression baculovirus vector by introducing an additional p6.9 promoter and an hr3 transcriptional enhancer into a polyhedrin promoter-based system and demonstrated, using reporter genes, that this design markedly increased recombinant protein expression [22]. Thus, applying such an enhanced BEVS platform to HPV vaccine manufacturing can improve production efficiency and lower manufacturing costs, thereby contributing to improved HPV vaccine access and reduced disease burden, particularly in developing countries.

Taken together, these considerations highlight the need for cost-effective, high-yield production platforms for next-generation multivalent HPV vaccines. Although currently licensed HPV vaccines are highly effective, they do not fully cover all oncogenic HPV types. Therefore, this study aimed to develop a 12-valent HPV vaccine with expanded type coverage, using an enhanced BEVS platform to improve production efficiency and industrial feasibility. We previously developed an enhanced baculovirus expression vector system, and in this study, we applied this platform to produce HPV L1 VLPs and evaluated its antigen yield and productivity. We also expanded the breadth of type coverage beyond that of the current 9-valent vaccine by including three additional high-prevalence HPV types (35, 39, and 59). Finally, we assessed the immunogenicity of the resulting 12-valent VLP formulation in mice to explore the feasibility of a vaccine candidate that offers antigenicity comparable to licensed products while being manufacturable at a lower cost.

## 2. Materials and Methods

### 2.1. Cells and Culture Medium

The *Spodoptera frugiperda* insect cell line (Gibco™ Sf9; Gibco, Grand Island, NY, USA) used for recombinant virus production was cultured in SF900™ III SFM, a serum-free medium (Gibco, Grand Island, NY, USA), at 27 °C with agitation at 130 rpm. Cells were sub-cultured every 7 days at a 20% working volume.

The *Trichoplusia ni* BTI-TN5B1-4 (High Five™, Hi5) insect cell line (Boyce Thompson Institute, Ithaca, NY, USA) used for protein expression was maintained in Express Five™ SFM (Gibco, Grand Island, NY, USA) at 27 °C with agitation at 130 rpm. Cells were sub-cultured every 3–4 days at a 10% working volume.

### 2.2. Construction of Recombinant Vectors and Viruses

Full-length HPV L1 gene sequences were obtained from the GenBank database (Supplementary Data S1). The C-terminal regions of the L1 genes that contain nuclear localization signals (NLSs) were truncated to enhance solubility and structural stability as previously described [22,23,24]. The modified HPV L1 sequences were codon-optimized for efficient expression in insect cells and synthesized by Bionics Inc. (Seoul, Republic of Korea) (Supplementary Data S1).

An enhanced expression transfer vector containing the polyhedrin promoter in combination with the p6.9 promoter, hr3, and repeated burst sequences was kindly provided by Professor Soo-Dong Woo (Chungbuk National University, Republic of Korea) [22,25]. The modified HPV L1 genes were cloned into the enhanced expression vector using the In-Fusion^®^ Cloning Kit (Takara Bio USA, Inc., San Jose, CA, USA) following the manufacturer’s instructions. Primer sequences and expected amplicon sizes for each HPV L1 gene are provided in Supplementary Data S1.

Recombinant baculoviruses were generated by co-transfecting the recombinant transfer vectors with flashBAC™ ULTRA baculovirus DNA (Oxford Expression Technologies Ltd., Oxford, UK) into Sf9 insect cells following the manufacturer’s protocol.

### 2.3. Expression and Purification

In this study, HPV L1 protein was expressed using rU-p6.9-HPV-L1 recombinant baculoviruses under optimal conditions established previously [23]. Briefly, 200 mL of Hi5 cells was prepared at a density of 2 × 10^6^ cells/mL in 1 L Erlenmeyer shaking flasks (Corning, NY, USA). Recombinant baculoviruses encoding HPV L1 were added at a multiplicity of infection (MOI) of 1, and the cultures were incubated at 27 °C and 130 rpm for 72 h.

Following incubation, cells were collected by centrifugation at 1000× *g* for 10 min and washed once with phosphate-buffered saline (PBS). Cell pellets were resuspended in lysis buffer composed of 20 mM Tris-HCl (pH 8.5) and 0.01% Tween-80 to a volume equivalent to the original culture. Cells were disrupted using LM20 Microfluidizer^®^ (Microfluidics, Westwood, MA, USA) with a single pass at 20,000 psi under chilled conditions. Cell debris was removed by centrifugation at 10,000× *g* for 20 min at 4 °C. The clarified extract was filtered through a 0.45 μm membrane filter unit (Corning, NY, USA) and immediately subjected to chromatographic purification using an ÄKTA go™ fast protein liquid chromatography system (Cytiva, Marlborough, MA, USA).

A two-step chromatographic purification procedure was used. First, the clarified lysate was subjected to anion-exchange chromatography using Source™ 30Q resin (Cytiva). Before loading, β-mercaptoethanol was added to promote capsid disassembly. Fractions containing L1 protein were identified by SDS-PAGE and immunoblotting and subsequently pooled. The pooled material was purified by hydroxyapatite chromatography (CHT-II resin, Bio-Rad, Hercules, CA, USA), and fractions containing the target protein were collected. The protein solution was concentrated using Amicon Ultra-15 centrifugal filter units (10 kDa MWCO, Millipore, Burlington, MA, USA) and desalted with a PD-10 column (Cytiva, Marlborough, MA, USA) equilibrated with reassembly buffer. Purified L1 protein was incubated at 4 °C to allow particle reassembly. Protein concentration was determined using a Bradford assay, and the final yield was calculated. The purity of the antigens was analyzed using the ‘plot lanes’ option in ImageJ 1.54g software (National Institutes of Health, Bethesda, MD, USA).

### 2.4. SDS-PAGE and Western Blot

HPV L1 proteins were analyzed by SDS-PAGE using 12% polyacrylamide gels, followed by Coomassie staining or transfer to PVDF membranes for Western blot analysis. For immunoblotting, membranes were probed with an HPV16 L1-specific monoclonal antibody (Camvir-1; Abcam, Cambridge, UK) followed by HRP-conjugated secondary antibodies, and signals were detected using WESTASVE ECL solution (AbFrontier, San Jose, CA, USA). Equal amounts of purified HPV L1 protein (4 μg per lane) were used for analysis to ensure consistent comparison across samples.

### 2.5. Dynamic Light Scattering (DLS)

Particle size distribution of HPV L1 VLPs was determined using a Zetasizer Ultra DLS system (ZSU5700, Malvern Panalytical Ltd., Malvern, Worcestershire, UK). A low-volume disposable sizing cell kit was used for measurements (ZSU1002, Malvern Panalytical Ltd., Malvern, Worcestershire, UK). Each sample was measured five times, and the mean number-weighted particle size was reported.

### 2.6. Transmission Electron Microscope (TEM)

TEM was performed to assess VLP formation of HPV L1 proteins. Carbon-coated copper grids (200 mesh) were glow-discharged at 25 mA for 30 s. Samples were applied to the grids for 4 min, followed by washing with deionized water and negative staining with 2% (*w*/*v*) uranyl acetate. A high-voltage transmission electron microscopy system (JEM-1400 Plus at 120 kV and JEM-1000BEF at 1000 kV; JEOL Ltd., Tokyo, Japan) at the Korea Basic Science Institute was used for imaging studies.

### 2.7. Immunization of Mice

The Institutional Animal Care and Use Committee of Optipharm Inc. approved the immunogenicity study (IACUC approval No. OPTI-IAC-2502). Eighty female 5-week-old BALB/c mice were randomly assigned to 16 groups (n = 5 per group) and acclimatized for seven days before experimentation. Mice were immunized with GARDASIL^®^9 (positive control); monovalent VLPs representing HPV types 6, 11, 16, 18, 31, 33, 35, 39, 45, 52, 58, and 59; a 9-valent vaccine; a 12-valent vaccine; or PBS (adjuvant alone, negative control). All vaccines were formulated with Alhydrogel^®^ adjuvant (2% aluminum hydroxide gel; InvivoGen, San Diego, CA, USA) as an adjuvant. The HPV type composition and concentrations of the vaccines are summarized in Table 1. Product doses for the mouse immunization study were selected based on approximately one-tenth of the anticipated human clinical dose for our HPV vaccine candidate, considering the established type-specific antigen content of the licensed HPV vaccine GARDASIL^®^9. This approach modeled the relative human antigen exposure while remaining within a dose range commonly used in preclinical mouse studies. The immunization dose of each monovalent vaccine was matched to that of the positive control. For serotypes 35, 39, and 59, the dosing regimen was established in accordance with previously published studies from other groups [9]. The vaccines were administered intramuscularly three times at two-week intervals (weeks 0, 2, and 4). A total volume of 100 μL was injected per immunization, with 50 μL injected into each hind thigh. Blood samples were collected at week 0 before immunization and at week 6.

### 2.8. Enzyme-Linked Immunosorbent Assay (ELISA)

Sera were analyzed using commercially available type-specific HPV L1 IgG ELISA kits specific for each tested serotype (Alpha Diagnostic International Inc., San Antonio, TX, USA), following the manufacturer’s instructions. Briefly, antigen-coated plates were incubated with diluted serum samples, followed by washing and incubation with HRP-conjugated secondary antibodies. After adding the TMB substrate, the reaction was stopped, and absorbance was measured at 450 nm using a microplate reader.

### 2.9. Statistical Analysis

Statistical analyses were performed using one-way ANOVA followed by Tukey’s multiple comparisons test in GraphPad Prism (version 8.2.1). Differences were considered statistically significant at *p* < 0.05, with *p*-values designated as follows: ns, not significant (*p* ≥ 0.05); * *p* < 0.05; ** *p* < 0.01; *** *p* < 0.001; **** *p* < 0.0001.

## 3. Results

### 3.1. Construction of an Enhanced Vector and Generation of HPV L1 Recombinant Baculoviruses

**Figure 1:**
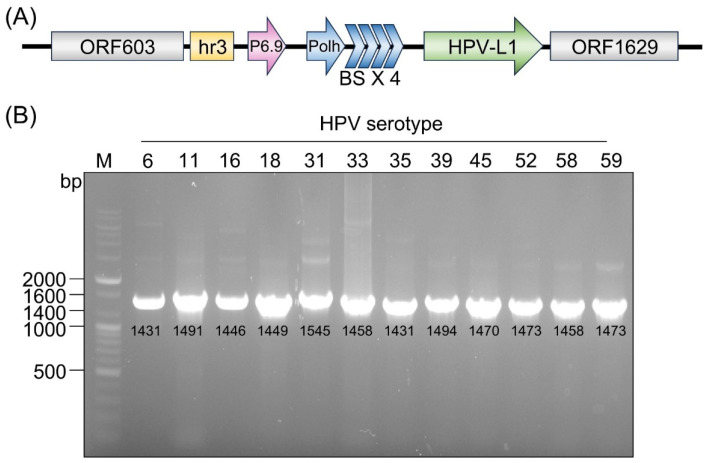
Construction of the HPV L1 enhanced vector and confirmation of recombinant HPV-L1 with PCR. (**A**) Schematic representation of the construction of the HPV L1 transfer vector containing the hr3 enhancer, p6.9 promoter, polyhedrin (Polh) promoter, and burst sequence (BS). (**B**) PCR analysis of HPV L1 genes (types 6, 11, 16, 18, 31, 33, 35, 39, 45, 52, 58, and 59) cloned into the transfer vectors. The expected PCR product sizes (bp) for each HPV L1 gene are indicated below each lane.

HPV L1 genes were cloned into an enhanced baculovirus expression vector (Figure 1). Recombinant baculoviruses encoding HPV L1 from serotypes 6, 11, 16, 18, 31, 33, 35, 39, 45, 52, 58, and 59 were generated using the flashBAC™ ULTRA system (Oxford Expression Technologies Ltd., Oxford, UK). HPV L1 genes were expressed in insect cells under the control of the p6.9 and polyhedrin promoters containing the hr3 and burst sequences following baculovirus infection. The presence of the HPV L1 genes in the recombinant baculoviral DNA was confirmed by polymerase chain reaction (PCR) using type-specific primers and by sequencing, as shown in Figure 1B.

### 3.2. Expression and Purification of HPV L1 Proteins

**Figure 2:**
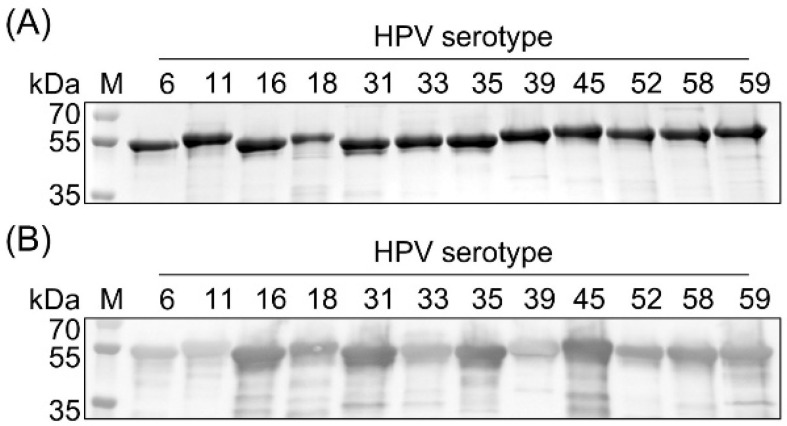
Purification of HPV L1 proteins produced in an enhanced baculovirus expression vector system. (**A**) SDS-PAGE analysis of purified HPV L1 proteins representing 12 HPV serotypes (6, 11, 16, 18, 31, 33, 35, 39, 45, 52, 58, and 59). (**B**) Western blot analysis of HPV L1 proteins using an anti-HPV16 L1 monoclonal antibody. M, molecular weight marker.

Hi5 insect cells were individually infected with 12 recombinant baculoviruses to express and purify HPV L1 proteins (Figure 2). Theoretical molecular weights and production yields of HPV L1 proteins for each serotype are summarized in Table 2. SDS-PAGE analysis of purified proteins at equivalent concentrations revealed a major band with consistent intensity at approximately 55 kDa, corresponding to the theoretical molecular weight of HPV L1, and all serotypes showed a purity of greater than 88% (Table 2). Under the same conditions, Western blot analysis showed some variation in band intensity among serotypes (Figure 2B), likely reflecting differences in cross-reactivity of the HPV16 L1-specific monoclonal antibody CamVir-1 toward individual L1 proteins, as previously reported [26,27]. The production yields of L1 proteins ranged from approximately 20 to 90 mg/L, depending on the serotype, highlighting the considerable commercial potential of this system for large-scale production of HPV L1 proteins. These differences in yield may be associated with variations in protein solubility and assembly efficiency, which can influence recovery during downstream purification.

### 3.3. Characterization of HPV L1 VLPs

**Figure 3:**
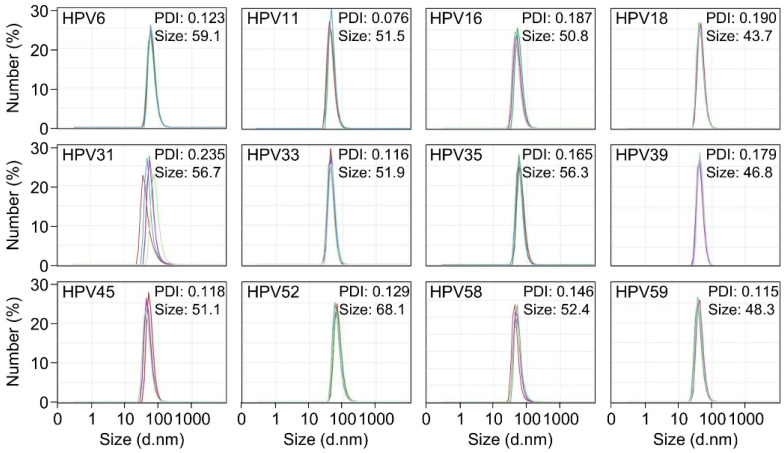
DLS analysis of HPV L1 VLPs. Number-weighted particle size distributions of HPV L1 VLPs from 12 HPV serotypes. The mean particle diameter and polydispersity index (PDI) are shown in each panel. Values represent the average of five measurements.

Formation and physicochemical properties of HPV L1 VLPs were evaluated by dynamic light scattering (DLS) and transmission electron microscopy (TEM). The hydrodynamic diameters of HPV L1 VLPs ranged from approximately 40 to 70 nm for all serotypes (Figure 3). The polydispersity index (PDI) values were below 0.3 for all preparations, indicating relatively homogeneous particle size distributions suitable for reliable DLS measurements [28].

**Figure 4:**
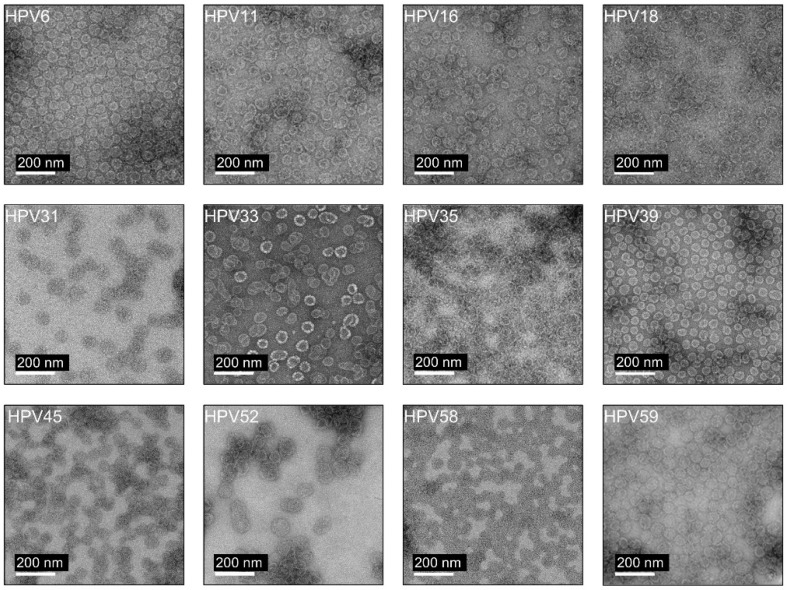
TEM analysis of HPV L1 VLPs. TEM images showing the morphology of HPV L1 VLPs assembled from purified L1 proteins representing twelve HPV serotypes. Images were collected from three randomly selected fields, and representative images are shown. Scale bars are indicated in white.

Consistent with the DLS results, negative-stain TEM revealed spherical, non-enveloped VLPs with diameters in the range of 40–70 nm and a uniform morphology across serotypes (Figure 4). Observed particle sizes were consistent with the reported diameter of native HPV virions (approximately 50–60 nm) [29]. These findings confirm that the recombinant HPV L1 proteins efficiently self-assembled into VLPs that closely resemble authentic HPV particles in size and morphology.

### 3.4. Immunogenicity of the 12-Valent HPV Vaccine in Mice

**Figure 5:**
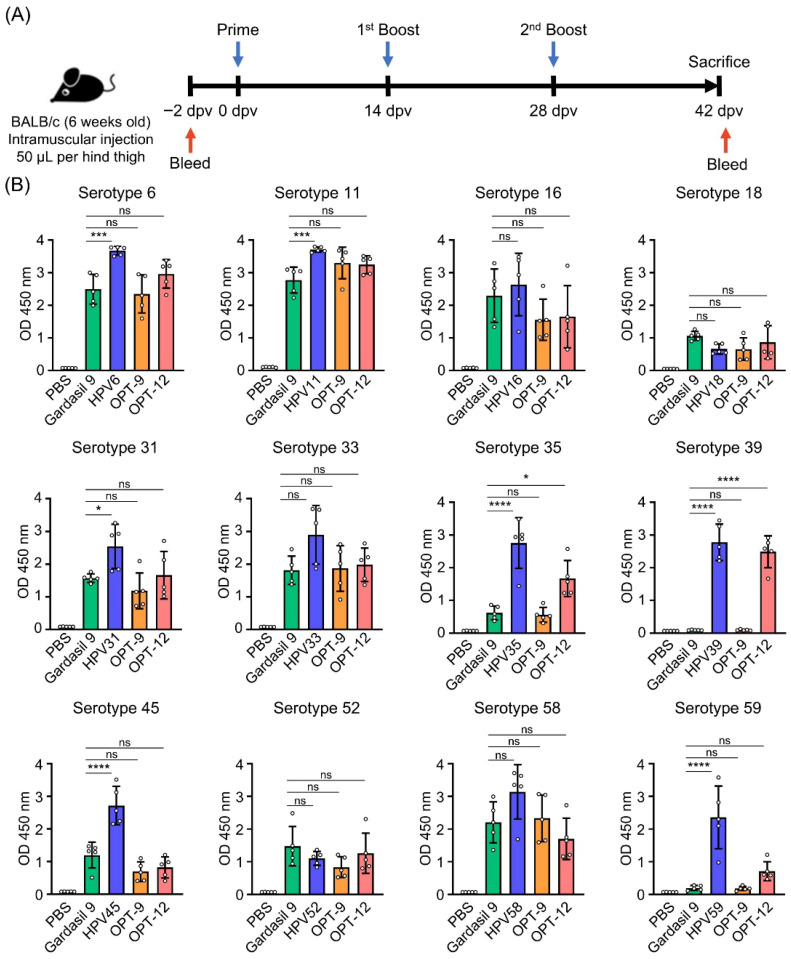
Immunogenicity of the vaccine candidates. (**A**) Schematic representation of the mouse immunization schedule. Briefly, female BALB/c mice were randomly divided into groups (n = 5 per group) and immunized intramuscularly with the vaccine candidates three times at two-week intervals. Serum samples were collected for analysis of HPV-specific IgG responses. (**B**) HPV-specific IgG antibodies were measured using a commercially available kit (containing HPV L1 antigen) following the manufacturer’s instructions. ns, not significant (*p* ≥ 0.05); * *p* < 0.05; *** *p* < 0.001; **** *p* < 0.0001.

A mouse study was conducted to evaluate the immunogenicity of the 12-valent HPV L1 VLP vaccine formulation (Figure 5A, Table 1). Monovalent vaccines corresponding to each HPV serotype were included as comparators to assess potential antigenic interference. Adjuvant alone was administered as a negative control, whereas GARDASIL^®^9 (9-valent) was used as a licensed positive control. An in-house 9-valent formulation (OPT-9) was prepared for direct comparison with GARDASIL^®^9 for the shared serotypes. No observable adverse effects, including allergic reactions, hypersensitivity, or mortality, were detected in any of the immunized mice during the study period.

Serum samples collected two weeks after the third immunization were analyzed for HPV type-specific IgG antibodies by ELISA (Figure 5B). For most serotypes, except for HPV-18 and HPV-52, the monovalent vaccine groups exhibited the highest antibody titers among all experimental groups. In contrast, antibody titers against HPV-18 and HPV-52 were comparable across vaccine groups, showing no statistically significant differences detected. Antibody responses did not differ between GARDASIL^®^9 and OPT-9 for any of the shared serotypes, and robust antibody titers were also induced against the three additional serotypes (HPV-35, HPV-39, and HPV-59) that are not included in GARDASIL^®^9. In contrast, low background signals were observed for non-shared serotypes in the OPT-9 and 12-valent groups, indicating minimal cross-reactivity and supporting the high specificity of the induced antibody responses. The monovalent vaccine groups showed the highest antibody titers. The 12-valent vaccine induced serotype-specific IgG responses against these additional types, which were observed in the GARDASIL^®^9 or OPT-9 groups, indicating that the 12-valent formulation can protect against a broader range of HPV serotypes.

## 4. Discussion

The HPV L1 major capsid protein self-assembles into VLPs that recapitulate the native virion structure, representing the principal antigenic component of current prophylactic HPV vaccines. In this study, we used an enhanced baculovirus expression vector system to produce 12 HPV L1 proteins and to evaluate their capacity to form VLPs. The baculovirus–insect cell platform is widely used to produce complex recombinant proteins and VLPs, owing to its high expression levels, proper protein folding, and scalability. Consistent with previous reports of hyper-expression baculoviral vectors, the use of a p6.9 promoter combined with an hr3 enhancer enabled the generation of HPV L1 VLPs with high production yields, ranging from approximately 20 to 90 mg/L depending on the serotype [22].

Production yields varied up to fourfold among serotypes, which may reflect differences in the solubility of L1 proteins after microfluidizer-based extraction. Amino acid sequence differences, particularly in the N-terminal or C-terminal regions, can markedly influence L1 expression, solubility, and VLP assembly efficiency in baculovirus systems [23,24]. In our system, the yields of HPV 6, 11, 16, and 18 L1 (approximately 40 mg/L) were comparable to those obtained with previously described hyper-expression vectors [30]. Furthermore, for the remaining serotypes, the production yields were comparable to or higher than the approximately 10 mg/L typically reported for many conventional HPV L1 VLP preparations [24,31,32]. Some optimized systems can achieve yields of approximately 30–50 mg/L, depending on the HPV serotype and production conditions [33,34]. In comparison, the enhanced BEVS developed in this study achieved yields ranging from 20 to 90 mg/L across all 12 serotypes, representing an approximately 2–5-fold improvement in productivity. This enhancement is likely attributable to the synergistic effects of the p6.9 promoter, hr3 enhancer, and burst sequence incorporated into the expression vector. The variations in production yield observed among different HPV serotypes may reflect inherent differences in protein properties, such as solubility, folding efficiency, and VLP assembly behavior, which can ultimately affect recovery during purification. These findings support the suitability of the enhanced baculovirus expression system as a production platform for multivalent HPV L1 VLP vaccines.

The structural properties of purified HPV L1 proteins were characterized by DLS and TEM. Most HPV L1 VLP preparations exhibited relatively homogeneous size distributions, with hydrodynamic diameters in the range of approximately 43–68 nm and polydispersity index values below 0.3, consistent with monodisperse VLP populations [28]. Although the PDI value for HPV31 (0.235) remained within the generally acceptable range (<0.3), it was higher than that of the other serotypes, indicating relatively increased heterogeneity in particle size distribution. Consistent with this observation, TEM analysis revealed the presence of smaller particles that were not prominently detected by DLS. Since SDS-PAGE analysis under denaturing conditions showed no significant impact on protein purity, these smaller particles are unlikely to result from protein degradation. They may represent partially assembled L1 structures or capsomeric forms rather than fully assembled VLPs. Negative stain TEM further confirmed the presence of spherical, non-enveloped VLPs, verifying that the L1 proteins assembled into particles with the expected morphology. For HPV 52, somewhat larger particles were observed compared with the other types, with an average particle size of 68.1 nm in DLS. This was attributed to subtle structural differences arising during self-assembly or to partial particle aggregation. HPV L1 proteins can form VLPs of varying diameters depending on the expression system, truncation strategy, and purification conditions, and such heterogeneity is considered an intrinsic feature of L1-based VLP assembly [35]. Overall, the particle sizes observed in this study are broadly consistent with the reported 50–60 nm diameter of native HPV virions and recombinant L1 VLPs.

In a mouse model, the 12-valent HPV L1 VLP vaccine elicited robust type-specific IgG responses against all included serotypes. Notably, the vaccine induced clear antibody responses to HPV 35, 39, and 59, which are not covered by currently licensed 9-valent formulations. Although antibody titers for some serotypes were modestly reduced in the multivalent formulation compared with the corresponding monovalent vaccines, this trend is consistent with the antigenic interference phenomenon described for other multivalent vaccines and is generally considered acceptable when overall coverage is expanded [36,37]. Importantly, for serotypes shared with licensed formulations, the 12-valent vaccine demonstrated IgG responses comparable to those induced by GARDASIL^®^9 and the in-house 9-valent formulation (OPT-9), suggesting that inclusion of additional antigens did not substantially compromise immunogenicity against the original target types. These data indicate that the 12-valent vaccine candidate can maintain immunogenicity similar to that of currently licensed 9-valent vaccines while broadening antigenic coverage.

In addition to GARDASIL^®^9, the development of multivalent HPV vaccines targeting more than nine oncogenic serotypes is currently underway, particularly in China [9,23,38]. In this study, we successfully produced a 12-valent HPV L1 VLP formulation and demonstrated that it elicited robust IgG responses in mice without apparent serotype-specific interference. Future work should therefore include a comprehensive pseudovirion-based neutralization assay (PBNA) for each serotype to confirm neutralizing activity, as well as longitudinal studies to evaluate the durability of antibody responses. Optimization of antigen dose and adjuvant formulation, along with a quantitative assessment of antigen–antigen interactions within highly multivalent formulations, will be important for minimizing antigenic interference while maximizing breadth of protection.

The stability of VLPs represents a critical parameter in vaccine development, as it directly influences antigenicity, storage conditions, and overall product quality. In this study, the reassembled HPV L1 VLPs retained their structural integrity during storage at 4 °C throughout the experimental period, indicating acceptable short-term stability. Nevertheless, a systematic assessment of the long-term stability of the VLPs under various storage and formulation conditions is warranted.

Finally, we designed a 12-valent antigen composition that extends the type coverage beyond that of the currently licensed 9-valent HPV vaccines. While existing 9-valent formulations provide substantial protection against the most common oncogenic HPV types, several high-risk genotypes remain unaddressed. In particular, HPV 35, 39, and 59 have been classified as carcinogenic to humans or associated with an increased risk of high-grade cervical lesions in epidemiological studies and IARC. By incorporating these three additional high-risk types, the 12-valent vaccine candidate has the potential to reduce the burden of HPV-associated disease. Realistically, achieving 100% prevention of HPV-associated disease is challenging; nevertheless, various strategies are being explored to further broaden type coverage, and vaccine candidates, including 14-valent or higher formulations, are already under investigation. Increasing vaccine valency inevitably introduces challenges related to manufacturing complexity and production costs. However, our results demonstrate that the enhanced baculovirus expression vector system can achieve relatively high yields for multiple HPV L1 antigens, providing a practical foundation for the development of next-generation multivalent HPV VLP vaccines.

## 5. Conclusions

In summary, this study demonstrates that diverse HPV L1 proteins expressed using an enhanced baculovirus expression vector system can reliably self-assemble into structurally intact VLPs. A 12-valent antigen formulation, incorporating additional high-risk HPV types not covered by current 9-valent vaccines, can maintain robust immunogenicity in a preclinical mouse model. Furthermore, the relatively high VLP yields achieved with this system may help mitigate the increased manufacturing complexity associated with multivalent formulations and provide a practical basis for lowering production costs and supporting cost-effective vaccine supply at the population level.

## Figures and Tables

**Table 1:** Vaccine candidates used to immunize mice in this study.

Group	Treatment	Serotype	L1 Dose μg/100 μL
1	PBS		-
2	GARDASIL^®^9	6, 11, 16, 18, 31, 33, 45, 52, and 58	27 μg
3	HPV6	6	3 μg
4	HPV11	11	4 μg
5	HPV16	16	6 μg
6	HPV18	18	4 μg
7	HPV31	31	2 μg
8	HPV33	33	2 μg
9	HPV35	35	2 μg
10	HPV39	39	2 μg
11	HPV45	45	2 μg
12	HPV52	52	2 μg
13	HPV58	58	2 μg
14	HPV59	59	2 μg
15	OPT-9	6, 11, 16, 18, 31, 33, 45, 52, and 58	27 μg
16	OPT-12	6, 11, 16, 18, 31, 33, 35, 39, 45, 52, 58, and 59	33 μg

Baseline blood samples were collected from each mouse two days before the first immunization (day −2). Vaccine formulations were administered intramuscularly at a dose of 100 μL per hind thigh on days 0, 14, and 28 at two-week intervals. Final blood samples were obtained on day 42.

**Table 2:** Purity and productivity of HPV L1 proteins.

Serotype	Predicted Size (kDa)	Purity (%)	Productivity (mg/L)
HPV6	51.9	99.3	67
HPV11	54.0	97.1	51
HPV16	52.6	97.9	25
HPV18	52.7	92.0	29
HPV31	56.4	94.6	90
HPV33	53.2	88.3	25
HPV35	52.1	98.8	45
HPV39	54.4	98.4	32
HPV45	53.6	98.8	61
HPV52	53.7	96.3	39
HPV58	53.5	97.9	22
HPV59	53.8	88.1	50

Predicted molecular weights were calculated using the SnapGene 8.2.2 software. Protein purity was determined by densitometric analysis using the ImageJ “plot lane” function. Productivity was calculated as the final purified protein yield, quantified by the Bradford assay, divided by the initial culture volume.

## Data Availability

All data used during the study are available from the corresponding author upon request.

## Supplementary Materials

The following supporting information can be downloaded at: https://www.mdpi.com/article/10.3390/vaccines14050398/s1, Supplementary Data S1: Codon-optimized nucleotide sequences of HPV L1 genes used in this study, Table S1: Summary of truncation design for HPV L1 constructs used in this study, Table S2: Primer sequences and expected PCR amplicon sizes for HPV L1 genes.

## Abbreviations

The following abbreviations are used in this manuscript:

HPV	Human papillomavirus
VLP	Virus-like particle
BEVS	Baculovirus expression vector system
BS	Burst sequence
ELISA	Enzyme-linked immunosorbent assay
SDS-PAGE	Sodium dodecyl sulfate–polyacrylamide gel electrophoresis
IACUC	Institutional Animal Care and Use Committee
CHT	Ceramic hydroxyapatite
DLS	Dynamic light scattering
IARC	International Agency for Research on Cancer
LMICs	Low- and middle-income countries
MOI	Multiplicity of infection
MWCO	Molecular weight cut-off
NIPs	National immunization programmes
NLS	Nuclear localization signal
PDI	Polydispersity index
TEM	Transmission electron microscopy
WHO	World Health Organization
Polh	Polyhedrin promoter
hr3	Homologous region 3 enhancer
OPT-9	9-valent HPV-L1 VLP vaccine in this study
OPT-12	12-valent HPV-L1 VLP vaccine in this study
ORF	Open reading frame
PBNA	Pseudovirion-based neutralization assay
