# Supplementary material for: Development of a 12-Valent HPV L1 Virus-like Particle Vaccine Using an Enhanced Baculovirus Expression System
Source: Vaccines (Basel). 2026 Apr 29;14(5):398. doi: 10.3390/vaccines14050398 (PMC13211534; doi:10.3390/vaccines14050398)
Supplement: Supplementary file 1 [file vaccines-14-00398-s001.zip › vaccines-4246930-supplementary.pdf]

Supplementary Data S1. Codon-optimized nucleotide sequences of HPV L1 genes used in this study

>HPV6-L1-codon optimization

ATGTGGCGCCCTAGCGACAGCACCGTATACGTGCCTCCTCCTAACCCTGTCTCCAAGGTTGTTGCCA
CGGACGCTTATGTTACCCGCACCAACATCTTCTACCACGCCTCGAGTTCTAGACTCCTTGCAGTCGG
ACATCCTTACTTTTCCATTAAGAGGGCTAATAAAACTGTTGTACCAAAAGTGTCAGGCTACCAGTAC
AGAGTCTTCAAAGTGGTGTTACCAGATCCAAACAAGTTTGCTCTGCCTGACTCCTCTTTGTTCGATC
CAACGACACAACGTCTGGTGTGGGCATGCACTGGTCTAGAGGTCGGGAGGGGCCAACCATTGGGC
GTGGGTGTATCAGGACACCCTTTCCTGAATAAGTACGACGATGTTGAAAACTCAGGTAGTGGTGGA
AACCCTGGACAGGACAACCGTGTCAACGTCGGCATGGATTATAAGCAAACACAGCTCTGCATGGTG
GGATGTGCGCCGCCTTTGGGCGAGCACTGGGGTAAGGGTAAACAATGTACTAATACACCTGTACAG
GCCGGTGACTGCCCGCCCTTGGAGCTCATCACCAGTGTAATCCAGGATGGTGATATGGTTGACACCG
GCTTCGGTGCTATGAACTTCGCTGACCTCCAGACGAATAAATCAGACGTGCCTATCGACATATGTGG
CACGACATGCAAATATCCAGACTACCTGCAGATGGCGGCAGATCCATACGGTGATCGTTTGTTCTTCT
TCCTGCGTAAGGAACAGATGTTCGCCAGACATTTCTTTAACAGGGCTGGAGAAGTGGGGGAGCCAG
TGCCTGACACTCTTATCATTAAGGGTAGCGGAAACCGCACGTCGGTAGGCTCTTCGATCTATGTCAA
CACCCCCAGCGGCTCACTGGTGTCCAGCGAAGCTCAACTCTTTAACAAGCCTTACTGGCTACAAAA
GGCCCAGGGTCACAACAATGGAATTTGCTGGGGAAATCAACTGTTCGTTACTGTCGTCGATACTACA
CGTAGTACCAACATGACATTATGTGCCTCCGTCACTACATCTTCCACCTACACCAACTCAGACTACAA
GGAGTACATGCGCCACGTGGAAGAATACGATCTGCAGTTCATCTTCCAGCTGTGCAGCATCACCCTA
AGCGCGGAGGTGATGGCATACATTCATACAATGAATCCTTCTGTTCTTGAGGACTGGAACTTCGGCT
TATCGCCGCCACCAAACGGTACGCTCGAAGACACCTACAGATACGTGCAATCACAGGCCATCACCT
GTCAAAAGCCCACTCCCGAAAAGGAGAAACCAGACCCCTACAAGAACCTGTCGTTTTGGGAGGTA
AATCTGAAGGAGAAGTTCTCTAGTGAACTCGATCAGTATCCTTTGGGACGCAAATTCTTGTTGCAAT
AA

>HPV11-L1-codon optimization

ATGAGCGACAGCACTGTGTACGTGCCCCCTCCCAACCCTGTATCCAAGGTTGTAGCCACGGACGCC
TACGTGAAACGCACCAACATCTTCTACCACGCTTCGAGTTCAAGACTGCTTGCTGTGGGACACCCAT
ACTACTCTATTAAGAAGGTGAACAAGACTGTTGTACCAAAGGTATCCGGGTACCAGTATAGAGTCTT
CAAAGTGGTTTTGCCAGATCCGAACAAGTTTGCACTGCCGGATTCATCTCTGTTCGACCCCACTACA
CAGCGTCTGGTATGGGCGTGCACAGGATTGGAGGTGGGCAGGGGTCAACCTCTCGGAGTCGGTGTG
TCGGGACACCCATTGCTCAACAAATATGACGATGTTGAGAATAGTGGTGGATATGGTGGCAATCCCG
GCCAGGACAACCGTGTCAACGTTGGTATGGATTACAAACAAACCCAGCTATGTATGGTCGGCTGTGC
TCCTCCTTTAGGTGAACATTGGGGTAAGGGAACGCAATGCTCTAACACCTCCGTCCAAAATGGCGAT
TGCCCCCCGTTGGAGCTTATCACCTCCGTGATACAAGATGGCGACATGGTTGATACCGGATTCGGTG
CCATGAACTTCGCCGACTTACAGACCAATAAGTCGGACGTTCCCCTGGACATCTGTGGAACTGTCTG
CAAATACCCCGACTATTTGCAAATGGCGGCAGACCCTTATGGTGATAGGCTCTTCTTCTACTTGCGCA
AGGAGCAGATGTTTGCACGTCACTTCTTCAACCGCGCCGGAACGGTAGGGGAACCTGTGCCAGAC
GATCTGTTGGTCAAGGGAGGCAATAATAGATCATCTGTCGCTAGTAGTATCTATGTCCATACACCTAG
TGGCTCACTCGTGTCGAGCGAGGCTCAACTCTTTAACAAGCCATACTGGCTGCAGAAGGCTCAGGG
CCACAACAACGGTATTTGCTGGGGTAACCACCTGTTCGTTACTGTGGTAGACACCACTCGTAGCACA
AATATGACACTATGTGCGTCCGTGTCTAAATCCGCTACTTACACGAACTCTGACTACAAAGAATACAT
GCGCCACGTGGAGGAGTTCGACCTGCAGTTCATCTTCCAACTCTGTAGCATTACTCTTTCCGCAGAA
GTCATGGCTTATATCCATACCATGAACCCTAGTGTCTTGGAAGATTGGAACTTCGGTTTATCGCCGCC
CCCTAATGGTACACTGGAAGATACTTACAGATACGTACAGTCACAGGCGATCACCTGCCAGAAACCA
ACGCCTGAGAAGGAGAAGCAGGACCCCTACAAGGACATGTCCTTCTGGGAGGTTAACCTCAAAGA
GAAGTTTTCCTCTGAACTAGACCAGTTTCCACTGGGACGTAAATTCCTGTTGCAATCAGGATACAGG
GGCCGCACTTCTGCCAGGACGGGCATCAAGCGACCAGCTGTGAGCAAGCCCTCTTAA

>HPV16-L1-codon optimization

ATGTCTCTGTGGCTGCCTAGTGAGGCCACCGTGTACCTGCCTCCTGTCCCAGTGTCCAAGGTCGTGA
GCACCGATGAATACGTGGCTCGCACCAACATCTACTACCACGCTGGAACTTCCAGACTGCTGGCTGT
GGGACACCCCTACTTCCCCATCAAGAAGCCTAACAACAACAAGATCCTCGTGCCTAAGGTGAGCGG
TCTGCAATACAGGGTGTTCCGTATCTACCTCCCCGACCCCAACAAGTTCGGATTCCCTGATACCTCAT
TCTACAACCCAGACACACAGCGCCTGGTCTGGGCTTGCGTCGGTGTGGAAGTGGGTCGTGGACAG
CCACTCGGTGTGGGCATCAGTGGCCACCCTCTGCTGAACAAGTTGGATGACACAGAGAACGCTAGT
GCCTACGCTGCTAACGCTGGCGTGGATAACCGCGAGTGCATCTCTATGGACTACAAGCAAACCCAGT
TGTGCCTGATCGGTTGCAAGCCCCCTATCGGTGAACACTGGGGCAAGGGATCCCCTTGCAACAACG
TGGCTGTGACACCAGGTGACTGCCCCCCACTGGAACTGATCAACACCGTGATCCAAGATGGCGACA
TGGTGGATACCGGCTTCGGTGCTATGGACTTCACCACACTGCAGGCTAACAAGAGTGAAGTGCCTC
TGGATATCTGCACATCTATCTGCAAGTACCCTGACTACATCAAGATGGTGTCAGAGCCTTACGGCGA
CAGCCTGTTCTTCTACCTGCGTAGGGAGCAAATGTTCGTCAGACACCTGTTCAACCGCGCTGGCGCT
GTGGGAGAAAACGTGCCAGACGACCTGTACATCAAGGGCTCCGGTTCTACTGCTAACCTGGCCAGT
TCAAACTACTTCCCTACACCTAGTGGTTCTATGGTGACCTCCGATGCCCAAATCTTCAACAAGCCTTA
CTGGCTGCAAAGAGCTCAGGGCCACAACAACGGCATCTGCTGGGGTAACCAGCTGTTCGTGACTGT
GGTGGACACTACCCGCTCAACCAACATGAGCCTGTGCGCTGCCATCTCCACTTCAGAACCTACCTAC
AAGAACACCAACTTCAAGGAGTACCTGAGGCACGGTGAGGAATACGACCTGCAGTTCATCTTCCAG
CTGTGCAAGATCACCCTCACTGCCGACGTGATGAGCTACATCCACTCCATGAACTCCACTATCTTGG
AGGACTGGAACTTCGGTCTCCAACCACCTCCAGGAGGCACCTTGGAGGATACCTACCGTTTCGTCA
CAAGCCAGGCTATCGCTTGCCAGAAGCACACACCTCCAGCTCCCAAGGAAGATCCCTTGAAGAAGT
ACACTTTCTGGGAAGTGAACCTGAAGGAAAAGTTCTCTGCCGACTTGGATCAATTCCCCCTGGGAA
GAAAGTTCCTGCTGCAATAA

>HPV18-L1-codon optimization

ATGGCTTTGTGGCGCCCTTCCGACAACACCGTCTACCTGCCACCTCCTTCTGTCGCTAGAGTGGTGA
ACACAGATGATTACGTGACTCGCACAAGCATCTTCTACCACGCTGGCAGCTCTCGCCTCCTGACTGT
GGGTAACCCATACTTCAGGGTCCCTGCTGGTGGTGGCAACAAGCAGGATATCCCTAAGGTGTCCGCC
TACCAATACAGAGTGTTCAGGGTGCAGCTGCCTGACCCAAACAAGTTCGGTCTGCCTGATACTAGTA
TCTACAACCTGGAGACCCAGCGTCTCGTGTGGGCCTGCGCTGGAGTGGAAATCGGCCGTGGTCAGC
CCCTGGGTGTGGGCCTGAGTGGTCACCCTTTCTACAACAAGCTGGATGACACTGAAAGTTCCCACG
CCGCCACCTCCAACGTCTCTGAGGACGTGAGGGACAACGTGTCTGTGGATTACAAGCAGACACAG
CTGTGCATCTTGGGCTGCGCCCCTGCTATCGGAGAGCACTGGGCTAAGGGCACCGCTTGTAAGTCCC
GTCCTCTGTCCCAGGGCGACTGCCCCCCTCTCGAACTGAAGAACACCGTGTTGGAAGATGGTGATA
TGGTGGACACTGGATACGGTGCCATGGACTTCTCCACCTTGCAAGACACCAAGTGTGAGGTGCCAC
TCGACATCTGCCAGTCTATCTGCAAGTACCCTGATTACTTGCAAATGTCAGCTGATCCTTACGGTGAT
TCCATGTTCTTCTGCCTGCGCCGTGAGCAACTGTTCGCCCGCCACTTCTGGAACAGGGCTGGCACC
ATGGGTGATACTGTGCCTCAATCACTGTACATCAAGGGCACAGGTATGCGTGCTTCACCCGGCAGCT
GTGTGTACTCCCCCAGCCCATCCGGCTCTATCGTGACCTCTGACTCCCAATTGTTCAACAAGCCATAC
TGGCTGCACAAGGCTCAAGGTCACAACAACGGTGTCTGCTGGCACAACCAACTGTTCGTGACTGTG
GTGGATACCACCCGCAGTACCAACCTGACAATCTGTGCTTCTACTCAGTCCCCCGTGCCTGGTCAGT
ACGACGCTACCAAGTTCAAGCAGTACAGCCGCCACGTGGAGGAATACGACTTGCAGTTCATCTTCC
AATTGTGTACTATCACTCTGACCGCTGATGTCATGTCCTACATCCACTCAATGAACAGCAGTATCCTG
GAGGATTGGAACTTCGGTGTGCCCCCCCCTCCAACCACTAGTTTGGTGGACACATACCGCTTCGTGC
AATCTGTGGCTATCACCTGTCAGAAGGATGCTGCTCCTGCTGAGAACAAGGACCCCTACGACAAGC
TCAAGTTCTGGAACGTGGACCTGAAGGAGAAGTTCTCTCTCGACCTGGATCAATACCCCCTGGGAA
GGAAGTTCTTGGTGCAGTAA

>HPV31-L1-codon optimization

ATGTCTCTGTGGCGGCCTAGCGAGGCTACCGTCTACCTCCCACCTGTCCCTGTGTCTAAGGTCGTGA
GCACGGACGAATACGTGACCCGAACCAACATATACTACCACGCAGGCAGCGCCAGGCTGCTTACGG
TAGGCCACCCATATTACTCCATCCCTAAATCCGACAATCCCAAAAAGATAGTTGTCCCCAAGGTGTCC
GGATTACAATACAGGGTATTCCGCGTTCGTCTGCCGGATCCAAACAAATTCGGATTTCCTGATACATC
TTTTTATAACCCTGAAACGCAACGCTTAGTCTGGGCTTGCGTCGGTCTAGAAGTGGGTCGCGGGCAG
CCGTTAGGTGTAGGTATCTCCGGTCATCCCCTATTGAACAAATTTGACGACACTGAGAACAGCAACC
GTTATGCCGGAGGTCCGGGCACCGACAACAGGGAATGTATCAGCATGGATTATAAGCAAACACAAC
TGTGCTTACTCGGCTGCAAACCCCCTATTGGAGAACACTGGGGTAAGGGTTCCCCATGTTCCAACAA
TGCCATCACCCCCGGTGACTGCCCCCCATTAGAATTAAAGAACTCAGTCATCCAGGACGGGGACATG
GTGGACACAGGCTTCGGAGCGATGGATTTTACTGCTCTGCAGGACACTAAAAGTAATGTTCCTTTGG
ACATTTGCAACTCTATCTGCAAATACCCTGATTACCTTAAGATGGTTGCTGAGCCGTACGGCGATACC
CTGTTTTTTTATTTGCGTAGGGAACAGATGTTCGTAAGGCACTTCTTCAACAGATCAGGCACGGTTG
GTGAATCGGTCCCTACAGACCTCTATATCAAGGGCTCCGGTTCAACAGCGACTTTAGCTAACAGTAC
GTACTTTCCTACTCCCAGCGGCTCCATGGTTACTTCAGATGCGCAAATCTTTAACAAACCATATTGGA
TGCAACGTGCTCAGGGACACAACAATGGTATTTGTTGGGGCAACCAGCTTTTCGTTACTGTGGTGG
ACACCACGAGAAGTACCAATATGTCCGTTTGCGCTGCCATCGCCAACTCAGACACTACGTTTAAATC
GAGTAATTTTAAGGAGTACCTCAGACATGGCGAGGAATTTGACTTGCAGTTTATATTTCAGCTCTGCA
AAATCACATTATCTGCAGACATCATGACATACATTCACAGTATGAATCCTGCTATTCTGGAGGACTGG
AATTTTGGATTGACCACTCCGCCCTCAGGTTCTTTGGAGGATACCTACAGGTTTGTGACCTCACAGG
CCATTACATGCCAAAAAACCGCCCCCCAGAAGCCCAAGGAGGATCCATTCAAAGACTACGTATTTTG
GGAGGTGAATCTAAAGGAGAAGTTTAGCGCAGATTTGGATCAATTTCCACTGGGTCGCAAATTCCTC
TTACAGGCGGGATACAGGGCACGCCCGAAGTTTAAGGCGGGCAAGCGAAGTGCACCCTCCGCCTCT
ACCACTACACCAGCTAAGCGTAAGAAAACTAAAAAATAA

>HPV33-L1-codon optimization

ATGTCCGTTTGGCGGCCCAGCGAGGCCACGGTGTACCTGCCTCCGGTCCCTGTATCTAAGGTAGTCT
CAACTGATGAATACGTGTCTCGCACAAGCATCTATTATTATGCGGGAAGTTCCAGACTCCTGGCTGTG
GGCCACCCATACTTCTCTATCAAAAATCCTACGAACGCTAAGAAATTATTGGTGCCCAAAGTAAGCG
GCTTGCAATATCGCGTTTTCAGGGTCCGTTTACCAGATCCTAATAAATTTGGATTCCCTGACACCAGC
TTTTACAACCCTGATACCCAACGACTTGTGTGGGCATGTGTAGGCTTGGAGATAGGACGTGGGCAGC
CATTAGGCGTGGGCATATCGGGCCATCCGTTACTCAACAAGTTCGACGACACTGAAACCGGTAACA
AGTATCCTGGTCAGCCGGGTGCGGATAATAGGGAATGTTTATCCATGGATTACAAGCAGACACAGTT
ATGTTTACTTGGATGTAAGCCCCCAACAGGTGAACACTGGGGTAAGGGTGTTGCTTGTACTAATGCC
GCTCCAGCCAATGATTGCCCACCTTTGGAACTCATCAACACTATCATCGAGGATGGCGACATGGTGG
ACACAGGATTTGGTTGCATGGACTTTAAAACATTGCAGGCTAATAAAAGTGATGTGCCCATTGACAT
CTGCGGCTCGACCTGCAAGTACCCAGACTACCTCAAAATGACTAGTGAGCCTTACGGTGATAGTCTG
TTTTTCTTTCTTCGACGTGAGCAAATGTTCGTGAGACACTTCTTTAACAGGGCTGGTACCTTAGGCG
AGGCGGTCCCCGATGACCTGTACATCAAAGGAAGCGGAACTACGGCCTCAATTCAAAGCTCGGCTT
TCTTCCCCACGCCTAGTGGATCAATGGTTACCAGCGAGTCTCAGTTATTTAATAAGCCGTATTGGCTA
CAGCGTGCACAAGGCCATAATAACGGTATTTGCTGGGGCAACCAAGTATTCGTTACGGTGGTGGACA
CCACTCGCTCGACTAACATGACTCTATGCACACAGGTCACCAGTGACAGTACGTACAAGAATGAAA
ACTTCAAAGAGTACATCAGACACGTCGAGGAGTACGACCTACAGTTCGTTTTCCAACTGTGCAAAG
TTACCTTAACGGCAGAAGTCATGACATACATCCATGCTATGAATCCCGATATTCTGGAGGATTGGCAA
TTCGGGCTGACTCCTCCCCCCTCCGCCAGTCTGCAGGACACCTACAGGTTCGTGACCTCTCAGGCC
ATTACGTGCCAGAAGACCGTCCCGCCAAAAGAAAAGGAAGACCCCTTAGGTAAATACACCTTCTGG
GAAGTGGACCTCAAGGAGAAATTTTCAGCCGACTTAGACCAGTTTCCTTTGGGACGCAAGTTCTTA
CTCCAGGCCGGTCTCAAAGCGTAA

>HPV35-L1-codon optimization

ATGACTGTTTACCTGCCTCCTGTTAGCGTGAGCAAGGTCGTGAGCACAGACGAATACGTGACCCGTA
CCAACATCTACTACCACGCCGGTTCTAGCCGCCTGCTGGCCGTCGGCCACCCCTACTACGCCATCAA
GAAGCAGGACTCCAACAAGATCGCTGTCCCCAAGGTCTCCGGCCTCCAGTACCGTGTTTTCCGTGT
GAAGCTGCCAGACCCCAACAAGTTCGGCTTCCCTGACACTAGCTTCTACGACCCTGCCTCCCAGCG
TCTGGTCTGGGCCTGCACCGGTGTGGAAGTGGGCCGCGGCCAGCCCCTGGGAGTGGGTATCTCCGG
TCACCCTCTGCTGAACAAGCTGGACGACACTGAAAACAGCAACAAGTACGTGGGCAACAGCGGAA
CCGACAACCGTGAATGTATCAGCATGGACTACAAGCAGACACAGCTGTGTCTCATCGGCTGTCGCC
CCCCAATCGGTGAACACTGGGGCAAGGGCACACCTTGTAACGCCAACCAGGTGAAGGCCGGTGAG
TGCCCTCCACTGGAGCTGCTCAACACCGTGCTGCAGGACGGCGACATGGTGGACACCGGCTTCGGT
GCTATGGACTTCACTACCCTCCAGGCCAACAAGTCCGACGTCCCCCTGGACATCTGCTCCAGCATCT
GCAAGTACCCTGACTACCTCAAGATGGTTAGCGAGCCTTACGGCGACATGCTGTTCTTCTACCTGCG
CCGCGAGCAAATGTTTGTCCGCCACCTGTTCAACCGTGCTGGCACCGTGGGTGAGACAGTGCCCGC
CGACCTGTACATCAAGGGTACTACCGGTACACTCCCTTCTACATCATATTTTCCAACTCCATCAGGTT
CAATGGTCACATCAGACGCTCAGATTTTCAACAAACCTTACTGGCTGCAGCGTGCCCAAGGTCATAA
CAACGGCATCTGCTGGTCCAACCAACTCTTCGTGACTGTCGTGGACACCACCCGCTCCACTAACAT
GAGCGTTTGTAGCGCTGTGTCCACCAGCGACTCCACATACAAAAACGACAACTTCAAGGAGTACCT
GCGCCACGGAGAGGAGTACGATCTGCAGTTCATCTTCCAGCTGTGTAAGATCACACTGACCGCTGA
CGTCATGACCTACATCCACAGTATGAACCCATCCATCCTCGAAGATTGGAACTTCGGACTGACACCC
CCCCCAAGTGGAACTCTGGAGGATACCTACAGATACGTCACCTCTCAAGCCGTCACATGCCAAAAG
CCCAGCGCCCCCAAGCCCAAAGATGACCCTCTGAAAAACTACACCTTCTGGGAAGTTGACTTGAAG
GAGAAGTTCAGCGCTGACTTGGACCAATTTCCCCTGGGTAGGAAGTTCCTCCTGCAAGCTGGTTTG
AAGGCTTAA

>HPV39-L1-codon optimization

ATGATGGTGTACCTGCCTCCCCCTAGCGTGGCTAAGGTGGTGAACACCGACGACTACGTGACACGC
ACTGGCATCTACTACTACGCCGGCTCCAGCCGCCTGCTGACAGTGGGCCACCCATACTTCAAGGTGG
GCATGAACGGTGGTCGTAAGCAAGACATCCCTAAGGTGAGCGCCTACCAGTACCGCGTGTTCCGTG
TGACCCTGCCAGACCCTAACAAGTTCTCCATCCCTGACGCTTCCCTGTACAACCCTGAAACACAGC
GCCTGGTGTGGGCTTGCGTTGGTGTGGAGGTGGGACGCGGTCAGCCTCTCGGTGTGGGCATCTCAG
GCCACCCACTGTACAACCGTCAGGACGACACAGAAAACTCCCCCTTCTCCTCCACTACTAACAAGG
ACAGCCGCGACAACGTGAGCGTTGACTACAAGCAGACCCAGCTGTGCATCATCGGTTGTGTGCCCG
CTATCGGTGAACACTGGGGCAAGGGCAAGGCTTGTAAGCCAAACAACGTGTCAACCGGCGACTGT
CCTCCCCTGGAGCTGGTGAACACCCCAATCGAGGACGGTGACATGATCGACACCGGTTACGGTGCT
ATGGACTTCGGAGCCCTGCAGGAAACCAAGAGCGAAGTCCCTCTGGACATCTGCCAGTCCATCTGT
AAGTACCCTGACTACCTGCAGATGAGCGCTGACGTGTACGGTGACAGCATGTTCTTCTGCCTGCGCC
GTGAGCAGCTGTTCGCCCGCCACTTCTGGAACCGCGGTGGCATGGTGGGTGACGCTATCCCTGCCC
AGCTGTACATCAAGGGCGCTGACATCCGCGCCAACCCCGGTAGCTCCGTGTACTGTCCCTCCCCTAG
CGGCAGCATGGTGACATCTGACAGCCAGCTGTTCAACAAGCCTTACTGGCTCCACAAGGCTCAGGG
CCACAACAACGGTATCTGCTGGCACAACCAACTCTTCCTGACCGTGGTTGACACAACTCGCTCCAC
TAACTTCACACTGTCCACCTCAATCGAATCCTCCATCCCTTCCACTTACGACCCTAGCAAGTTCAAG
GAGTACACTCGCCACGTTGAAGAATACGACCTGCAGTTCATCTTCCAGCTGTGCACCGTGACACTG
ACCACTGACGTGATGAGCTACATCCACACCATGAACAGCTCCATCCTGGACAACTGGAACTTCGCC
GTGGCCCCTCCTCCAAGCGCTTCCCTGGTGGACACCTACCGCTACCTGCAGTCAGCCGCCATCACCT
GTCAGAAGGACGCTCCTGCTCCTGAGAAGAAGGACCCCTACGACGGCCTGAAGTTCTGGAACGTG
GACCTGCGTGAGAAGTTCTCCCTGGAACTGGACCAGTTCCCCCTGGGTCGTAAGTTCCTGCTGCAG
GCCCGTGTGCGCCGCCGTCCTACAATCGGCCCACGTAAGCGCCCCGCCGCCTCCACCTCCAGCAGC
AGCGCTTGA

>HPV45-L1-codon optimization

ATGGCCTTGTGGCGGCCTTCAGACAGTACGGTGTATCTTCCACCACCCTCTGTGGCCCGCGTCGTCA
GCACTGACGACTATGTGTCTCGCACCAGCATATTCTACCACGCAGGAAGTTCCAGATTACTCACTGT
TGGCAATCCATACTTCAGGGTTGTACCGAATGGTGCGGGTAACAAGCAGGCTGTGCCTAAGGTGTCC
GCGTACCAGTACAGGGTGTTTAGAGTAGCCTTACCCGACCCTAATAAATTTGGACTCCCTGATTCTAC
TATATATAATCCAGAAACACAACGTTTGGTTTGGGCATGCGTAGGTATGGAAATTGGTCGTGGTCAGC
CTCTCGGTATTGGCCTATCAGGCCACCCCTTCTATAACAAGTTGGATGATACAGAGAGTGCCCATGCC
GCTACAGCGGTTATCACGCAGGATGTTAGAGATAACGTGTCAGTGGACTACAAGCAAACCCAGCTG
TGCATTCTGGGTTGCGTTCCTGCTATAGGCGAGCACTGGGCCAAGGGCACCCTTTGTAAGCCTGCGC
AGTTGCAACCGGGTGACTGCCCTCCTTTGGAACTTAAAAACACGATTATTGAGGATGGAGACATGG
TGGACACAGGATATGGCGCAATGGACTTCAGTACACTGCAGGACACAAAATGCGAGGTTCCGCTCG
ACATCTGCCAATCCATCTGTAAATACCCGGATTACTTGCAAATGTCCGCTGACCCCTATGGGGACTCT
ATGTTTTTCTGCCTGCGCCGCGAGCAGCTGTTCGCAAGACACTTTTGGAACAGGGCCGGTGTAATG
GGTGACACTGTACCGACGGACCTATATATTAAAGGCACTTCAGCTAATATGCGTGAAACCCCAGGCA
GTTGTGTTTACTCCCCCTCGCCCTCGGGCAGCATTATTACGTCGGACTCTCAGCTGTTCAACAAGCC
ATATTGGCTCCATAAGGCTCAGGGCCACAACAATGGTATTTGCTGGCACAATCAGTTGTTTGTTACTG
TGGTGGACACAACGCGCTCAACCAATTTAACGCTCTGTGCCTCTACACAAAACCCGGTCCCAAGTA
CATATGACCCAACTAAGTTCAAGCAGTACAGTAGACACGTGGAGGAGTATGATTTACAGTTTATCTT
CCAGTTGTGCACCATTACTCTGACTGCAGAGGTGATGTCATACATCCATTCGATGAACAGTAGTATAC
TGGAAAACTGGAACTTTGGTGTCCCTCCACCACCGACTACCAGTCTGGTGGACACATATCGCTTCGT
GCAATCAGTCGCTGTTACCTGTCAGAAGGATACCACACCTCCAGAAAAGCAGGATCCCTACGATAA
ACTGAAGTTCTGGACTGTTGACCTGAAGGAAAAGTTCTCCAGCGATCTCGATCAATACCCCCTTGGT
CGAAAGTTCTTAGTCCAGGCTGGGTTGCGATAA

>HPV52-L1-codon optimization

ATGTCCGTCTGGCGGCCTTCCGAGGCCACGGTGTACCTGCCTCCCGTACCTGTCTCGAAGGTTGTAA
GCACTGACGAGTATGTGTCTCGCACAAGCATCTATTACTACGCAGGCTCTTCTCGACTCCTGACAGT
CGGACATCCCTATTTTTCTATTAAGAACACCTCCTCGGGTAACGGGAAAAAGGTTCTGGTTCCCAAG
GTGTCTGGCCTGCAATACAGGGTATTTAGAATTAAGTTGCCGGACCCCAACAAATTCGGATTCCCAG
ACACATCTTTTTATAACCCCGAGACCCAACGCTTGGTCTGGGCCTGTACAGGCTTGGAAATCGGTAG
GGGACAGCCCTTAGGTGTGGGTATCTCAGGCCATCCACTGCTCAACAAGTTCGACGACACGGAAAC
CAGTAACAAGTACGCTGGCAAACCTGGTATAGATAACAGGGAGTGCTTATCTATGGATTATAAGCAA
ACTCAGCTGTGCATTTTAGGATGCAAACCGCCTATCGGCGAACACTGGGGTAAGGGAACCCCGTGC
AACAACAATTCAGGAAATCCTGGCGATTGTCCTCCCCTACAACTCATTAACTCAGTAATACAGGACG
GCGACATGGTCGATACAGGATTCGGTTGCATGGACTTTAACACCTTGCAGGCTAGTAAGAGTGATGT
CCCCATCGATATCTGCAGCAGTGTGTGTAAGTATCCAGATTACTTGCAGATGGCTAGCGAGCCGTAC
GGCGACTCCTTGTTCTTTTTTCTTAGACGAGAGCAAATGTTTGTTAGACACTTCTTTAATAGGGCCGG
AACCCTCGGTGACCCCGTGCCAGGTGATCTGTATATCCAAGGGAGCAACTCGGGCAACACTGCCAC
TGTACAAAGCAGTGCGTTTTTTCCTACGCCTTCCGGTTCTATGGTAACCTCAGAATCCCAACTCTTCA
ACAAACCGTACTGGTTACAGCGTGCGCAGGGCCACAATAACGGCATATGTTGGGGAAATCAGTTGT
TCGTCACCGTTGTGGATACCACTCGTAGCACTAACATGACTCTTTGTGCGGAGGTGAAAAAGGAAA
GCACCTACAAAAATGAGAATTTCAAGGAATACCTGCGTCATGGCGAGGAATTCGACCTGCAGTTTAT
TTTTCAGCTTTGCAAGATCACACTGACAGCTGACGTTATGACGTACATTCACAAGATGGATGCCACT
ATTCTGGAGGACTGGCAATTTGGCCTTACCCCACCACCTTCAGCGTCATTGGAGGACACATACAGAT
TTGTGACCTCTACTGCCATCACTTGCCAAAAAAACACCCCACCGAAGGGAAAAGAGGATCCTCTGA
AGGACTACATGTTCTGGGAGGTGGACTTGAAAGAAAAGTTTTCTGCTGACCTCGACCAGTTCCCTT
TAGGTCGTAAGTTCTTGCTCCAGGCAGGGCTACAGGCTTAA

>HPV58-L1-codon optimization

ATGTCCGTGTGGCGGCCTAGTGAGGCCACTGTGTACCTGCCTCCCGTGCCTGTGTCCAAGGTTGTAT
CCACGGATGAATACGTGTCACGCACAAGCATTTATTATTACGCTGGCAGTTCCCGCCTTTTGGCTGTT
GGCAATCCATATTTCTCAATCAAGAGTCCTAACAACAATAAAAAGGTACTGGTTCCCAAGGTATCAG
GCTTACAGTACAGGGTCTTTCGCGTGCGTCTGCCTGATCCCAATAAATTCGGTTTCCCTGACACATCT
TTTTATAACCCTGACACGCAAAGACTCGTCTGGGCCTGTGTCGGACTGGAGATAGGTAGGGGACAG
CCATTGGGTGTCGGCGTATCGGGTCATCCTTATTTAAATAAATTCGATGACACTGAGACCAGTAACAG
ATATCCCGCACAGCCAGGTTCTGACAACAGGGAGTGCTTATCTATGGACTATAAACAGACACAACTG
TGCCTTATCGGCTGTAAACCTCCCACTGGTGAGCACTGGGGAAAAGGCGTTGCCTGTAACAATAAC
GCGGCTGCTACCGACTGTCCCCCTCTCGAACTCTTTAACTCTATAATCGAGGATGGTGACATGGTAG
ACACTGGGTTTGGATGCATGGACTTTGGTACATTGCAGGCTAATAAGAGTGACGTGCCTATCGATATT
TGTAACTCAACGTGCAAATACCCAGATTACCTGAAGATGGCCTCAGAACCCTATGGGGATAGTTTGT
TCTTCTTCCTCCGCCGTGAGCAGATGTTTGTGAGACACTTTTTCAACAGGGCTGGAAAGCTTGGCG
AAGCTGTCCCGGACGACCTTTATATTAAAGGATCCGGAAATACGGCAGTGATCCAATCGAGTGCATT
CTTCCCAACTCCGAGTGGTTCTATCGTTACCTCAGAGTCGCAACTCTTTAACAAGCCTTATTGGTTGC
AGCGTGCGCAAGGACATAACAACGGCATTTGCTGGGGCAACCAGTTATTCGTCACCGTGGTTGATA
CCACTAGGTCCACCAACATGACATTATGCACCGAAGTAACTAAGGAAGGTACGTATAAAAATGATAA
CTTCAAGGAGTATGTCCGTCACGTCGAAGAATATGACTTACAGTTCGTGTTCCAGCTCTGCAAGATC
ACACTAACTGCAGAGATCATGACATACATACACACTATGGATTCCAATATCTTGGAGGATTGGCAATT
CGGTCTGACACCGCCTCCGTCGGCCAGTTTACAGGACACATACAGATTCGTTACCAGCCAGGCTATC
ACCTGCCAGAAGACCGCTCCCCCGAAGGAGAAGGAAGATCCATTAAATAAGTATACTTTCTGGGAG
GTTAACCTCAAGGAAAAGTTCTCTGCGGACCTAGACCAATTTCCATTGGGACGAAAGTTTCTGTTAC
AATCAGGCCTTAAAGCTTAA

>HPV59-L1-codon optimization

ATGGCTCTCTGGCGTTCCAGCGACAACAAGGTGTACCTGCCACCTCCTAGCGTCGCCAAGGTTGTG
TCCACCGACGACTACGTGACTCGCACCAGCATCTTCTACCACGCCGGCAGCAGCCGCCTGCTCACC
GTGGGCCACCCTTACTTCAAGGTGCCTAAGGGTGGCAACGGCCGTCAGGACGTTCCTAAGGTGAGC
GCTTACCAGTACCGCGTGTTCCGCGTGAACCTGCCTGACCCCAACAAGTTCGGCCTGCCTGACAAC
ACAGTGTACGACCCTAACTCCCAGCGCCTGGTGTGGGCCTGCGTCGGCGTGGAAATCGGCCGTGGC
CAGCCACTGGGAGTGGGCCTCTCCGGTCACCCCTTCTACAACAAGCTGGACGACACAGAAAACTC
CCACGTCGCCAGCGCTGTGGACAACAAGGACACCCGTGACAACGTTTCCGTGGACTACAAGCAGA
CCCAGCTGTGTATCATCGGCTGCGTCCCCGCCATCGGTGAACACTGGACCAAGGGCACAGCCTGTA
AGCCAAACACTGTGGTGCAGGGTGACTGCCCCCCTCTGGAGCTGATCAACACCCCAATCGAAGATG
GCGACATGGTTGACACAGGATACGGTGCTATGGACTTCAAGCTGCTCCAGGACACCAAGTCCGAGG
TCCCCCTGGACATCTGCCAGAGCATCTGTAAGTACCCTGACTACCTCCAGATGAGCGCTGACGCCTA
CGGTGACTCCATGTTCTTCTGCCTGCGTCGCGAACAGGTGTTCGCTCGTCACTTCTGGAACCGCTCC
GGTACTATGGGTGACCAAATCCCAGAATCCCTGTACATCAAGGGCACCGACATCCGCGCCACCCCCG
GTTCCTACCTGTACAGCCCCTCCCCAAGCGGTTCCGTGGTGACATCCGACTCCCAACTGTTCAACAA
GCCTTACTGGCTGCACAAGGCCCAGGGCCTGAACAACGGTATCTGTTGGCACAACCAACTCTTCCT
GACCGTGGTTGACACCACTCGCAGCACTAATCTCTCCGTGTGCGCTTCCACAACCAGCAGCATCCCT
AACGTGTACACCCCCACATCCTTCAAGGAATACGCTCGCCACGTTGAGGAGTTCGACCTCCAGTTC
ATCTTCCAACTGTGCAAGATCACACTGACTACAGAGGTTATGTCCTACATCCACAACATGAACACCA
CCATCCTGGAGGACTGGAACTTCGGTGTGACTCCTCCTCCAACAGCCTCTCTCGTGGACACCTACC
GCTTCGTGCAGAGCGCCGCTGTGACTTGTCAGAAGGACACTGCTCCTCCCGTGAAGCAGGACCCTT
ACGACAAGCTGAAGTTCTGGACTGTGGACCTGAAGGAGCGCTTCAGCGCTGACCTGGACCAGTTC
CCTCTGGGTCGCAAGTTCCTCCTGCAGCTGGGCGCCCGTCCTAAGCCCACCTAA

Table S1. Summary of truncation design for HPV L1 constructs used in this study

HPV-L1 type	Full length (aa)	Construct (aa range)	Truncation Type
HPV6	517	1-466	C-terminal
HPV11	501	5-489	N,C-terminal
HPV16	531	28-497	N,C-terminal
HPV18	568	63-533	N,C-terminal
HPV31	504	1-504	-
HPV33	499	1-475	C-terminal
HPV35	502	10-474	N,C-terminal
HPV39	505	10-495	N,C-terminal
HPV45	539	28-505	N,C-terminal
HPV52	529	28-506	N,C-terminal
HPV58	524	28-501	N,C-terminal
HPV59	508	1-480	C-terminal

Table S2. Primer sequences and expected PCR amplicon sizes for HPV L1 genes

HPV type	Reverse primer (5'->3')	Reverse primer (5'->3')	Length (bp)
HPV6	ATAAATAGCTAGCCCATGTGGCGCC CTAGC	CGCATGCCTCGAGACTTATTGC AACAAGAATTTGCGTCC	1431
HPV11	ATAAATAGCTAGCCCATGAGCGAC AGCACTGTGTAC	CGCATGCCTCGAGACTTAAGA GGGCTTGCTCACAG	1491
HPV16	ATAAATAGCTAGCCCATGTCTCTTT GGCTGCCGA	GATTCGAAAGCGGCCTTATTGT AGTAAAAATTTCTTCCTAAGG GG	1446
HPV18	ATAAATAGCTAGCCCATGGCGTTGT GGCGG	GATTCGAAAGCGGCCTTACTG AACCAAAAATTTCCTTCCAAG G	1449
HPV31	ATAAATAGCTAGCCCATGTCTCTGT GGCGGCC	GATTCGAAAGCGGCCTTATTTT TTAGTTTTCTTACGCTTAGCTG GTG	1545
HPV33	ATAAATAGCTAGCCCATGTCCGTTT GGCGGC	GATTCGAAAGCGGCCTTACGC TTTGAGACCGGC	1458
HPV35	ATAAATAGCTAGCCCATGACTGTTT ACCTGCCTCC	GATTCGAAAGCGGCCTTAAGC CTTCAAACCAGCTTGC	1431
HPV39	ATAAATAGCTAGCCCATGATGGTGT ACCTGCCTCC	GATTCGAAAGCGGCCTCAAGC GCTGCTGCTGG	1494
HPV45	ATAAATAGCTAGCCCATGGCCTTGT GGCGG	GATTCGAAAGCGGCCTTATCGC AACCCAGCCTG	1470
HPV52	ATAAATAGCTAGCCCATGTCCGTCT GGCGG	GATTCGAAAGCGGCCTTAAGC CTGTAGCCCTGCC	1473
HPV58	ATAAATAGCTAGCCCATGTCCGTGT GGCGG	GATTCGAAAGCGGCCTTAAGC TTTAAGGCCTGATTGTAAC	1458
HPV59	ATAAATAGCTAGCCCATGGCTCTCT GGCGTTCC	GATTCGAAAGCGGCCTTAGGT GGGCTTAGGACGG	1473
